# Clinical management of a severe pressure injury with Buzhong Yiqi Decoction in the ICU: A case report

**DOI:** 10.1097/MD.0000000000051226

**Published:** 2026-10-30

**Authors:** Dan Liao, Bangjiang Fang, Jianjian Li, Jing Wang, Wei He, Xiaosong Lyu, Yan Zhang

**Affiliations:** a Jiangjin Hospital, Chongqing University of Chinese Medicine, Chongqing, People’s Republic of China; b Intensive Care Unit, Chongqing Jiangjin District Hospital of Chinese Medicine, Chongqing, People’s Republic of China; c Department of Emergency Medicine, Longhua Hospital, Shanghai University of Traditional Chinese Medicine, Shanghai, People’s Republic of China; d Department of Acupuncture and Moxibustion, Chongqing Jiangjin District Hospital of Chinese Medicine, Chongqing, People’s Republic of China.

**Keywords:** case report, pressure injuries, traditional Chinese medicine

## Abstract

**Rationale:**

Severe pressure injuries in long-term intensive care unit (ICU) bedridden patients are often combined with multidrug-resistant infections and life-threatening complications, including sepsis. Standard Western therapies such as debridement and biological dressings frequently fail to heal refractory wounds, triggering recurrent fever and substantial clinical and economic burdens. Traditional Chinese Medicine (TCM) offers an alternative adjuvant approach via holistic syndrome differentiation to regulate systemic function and accelerate wound granulation.

**Patient concerns:**

A 60-year-old ICU male with severe pneumonia and type I respiratory failure developed a stage IV pressure injury. After 6 months of standardized Western interventions, including antibiotics, surgical debridement, and pressure-relief nursing, the wound worsened with persistent recurrent fever caused by multidrug-resistant pathogens.

**Diagnoses:**

Western diagnosis: stage IV pressure injury. TCM diagnosis: decubital wound with spleen deficiency and sunken qi complicated by phlegm-stasis-damp-toxin obstruction.

**Interventions:**

Modified Bu Zhong Yi Qi Tang was administered to tonify middle jiao, lift sunken yang, consolidate vital qi, and promote granulation.

**Outcomes:**

Continuous herbal treatment for over 20 days achieved complete wound healing, resolved fever, and allowed successful discontinuation of mechanical ventilation and antibiotics. The patient’s appetite and mental status improved remarkably without adverse hepatic or renal reactions. Notably, full recovery relied on combined Western supportive care rather than herbal medicine alone.

**Lessons:**

This TCM regimen may represent a potential adjunctive therapy for refractory ICU stage IV pressure injuries unresponsive to routine Western management. The favorable outcome resulted from integrated Chinese-Western multimodal treatment. Further large prospective trials are required to verify the independent efficacy of Modified Bu Zhong Yi Qi Tang.

## 1. Introduction

Pressure injuries result from damage to local skin and underlying tissues caused by intense or prolonged pressure, or the combined effect of pressure and shear forces. They are more prevalent in elderly patients with prolonged bed rest and individuals with limited mobility, most commonly occurring at bony prominences or mucosal sites compressed by medical devices.^[1,2]^ According to the guidelines released by the National Pressure Injury Advisory Panel and European Pressure Ulcer Advisory Panel, pressure injuries can be categorized into stages I to IV, suspected deep tissue injuries, and unstageable. In severe cases, pressure injuries may be complicated by multidrug-resistant bacterial infections, which can further progress to life-threatening complications such as necrotizing fasciitis, abscesses, and sepsis, thereby endangering patients’ lives.^[3]^ Patients in the intensive care unit (ICU) face high-pressure injury risks due to critical illness and prolonged bed rest. ICU pressure injury incidence may reach 18% to 39%,^[4]^ and various critical illness-related factors further drive early deep tissue pressure injuries.^[5]^ Currently, Western treatments, including debridement, growth factors, cell therapy, and biological dressings, often fail to achieve satisfactory wound healing in ICU patients with multidrug-resistant infections, who suffer from persistent fever and prolonged hospitalization, which constitutes a critical clinical dilemma.

Despite accumulating studies confirming the wound-repair effects of traditional Chinese medicine (TCM) for pressure injuries, most existing literature focuses on mild-to-moderate lesions or simple topical therapies.^[6]^ Few published case reports specifically focus on stage Ⅳ ICU-acquired pressure injuries accompanied by recurrent multidrug-resistant infections and persistent qi deficiency. There remains a lack of clinical evidence describing the full-course internal administration of Modified Bu Zhong Yi Qi Tang for such refractory, life-threatening wounds, which creates an obvious research gap worthy of further exploration.

Professor Fang Bangjiang, a “Qihuang Scholar” and national TCM academic inheritance instructor, has conducted decades of clinical research on refractory pressure injuries and formed unique syndrome differentiation ideas for critically ill patients. In TCM theory, pressure injuries arise from prolonged local pressure inducing qi stagnation and blood stasis, coupled with insufficient qi and blood to nourish skin and muscle tissues, ultimately leading to tissue necrosis and ulcer formation.^[7]^ Critically ill ICU patients suffer severe consumption of primordial qi due to severe primary disease, long-term mechanical ventilation, and repeated infections, resulting in concurrent spleen deficiency and sunken qi – the core pathogenesis of intractable pressure injuries in this population. Internal TCM treatment targeting the replenishment of middle jiao and lifting sunken yang can simultaneously regulate systemic deficiency and promote local granulation growth, showing unique advantages that complement Western wound care.

This case report describes the comprehensive management of a patient with a severe ICU-acquired stage IV pressure injury combined with multidrug-resistant bacterial infections under Professor Fang Bangjiang’s care, with Modified Bu Zhong Yi Qi Tang administered as part of the integrated treatment regimen and encouraging wound-healing outcomes. We hereby report this case to supplement clinical observations of internal TCM adjuvant therapy for refractory ICU pressure injuries and provide a potential auxiliary treatment strategy for similar intractable wounds.

## 2. Case report

### 2.1. Clinical information

The patient was a 60-year-old male admitted to the Respiratory Department of Chongqing Jiangjin District Hospital of Traditional Chinese Medicine on December 30, 2022, presenting with “cough, exertional dyspnea and fever for 1 week, aggravated over the past day.” On the second day after admission, the patient’s exertional dyspnea deteriorated accompanied by low oxygen saturation, and he was transferred to the ICU for monitoring and treatment. During hospitalization, the patient developed recurrent high fever with a peak temperature of 40°C. Multiple bronchoalveolar lavage fluid cultures isolated *Acinetobacter baumannii* complex/*Acinetobacter haemolyticus*, *Pseudomonas aeruginosa*, *Escherichia coli*, *Candida albicans*, *Enterobacter cloacae*, and *Klebsiella pneumoniae*, all of which were multidrug-resistant. Chest computed tomography (CT) and laboratory examinations confirmed severe pneumonia and type I respiratory failure. The patient underwent tracheal intubation followed by tracheostomy, with invasive mechanical ventilation to improve oxygenation. Antimicrobial therapy included piperacillin-tazobactam, meropenem, imipenem, cefoperazone-sulbactam, co-trimoxazole, levofloxacin, fluconazole, and vancomycin for anti-infective therapy. Concurrent symptomatic management included enhanced sputum drainage, mucolytic agents, and enteral nutrition via a nasogastric tube for nutritional support. The nursing team intensified daily care, maintaining skin cleanliness and dryness, performing regular repositioning, and using an air-cell mattress to prevent pressure injury. Due to the patient’s critical condition and poor respiratory function, extubation was not feasible. The patient underwent prolonged bed rest treatment, moving between the ICU and Pulmonary Medicine Department. With minimal spontaneous movement, a bowl-sized pressure injury gradually developed on the buttocks, measuring approximately 8 cm × 10 cm in diameter. This ulcer corresponds to stage IV pressure injury according to the guidelines published by the National Pressure Injury Advisory Panel and the European Pressure Ulcer Advisory Panel. The ulcer surface appeared pale and nonluminous, with exposed bone covered by foul-smelling, purulent secretions with a putrid odor. Sterile cultures of the pus yielded *K pneumoniae*, *Staphylococcus aureus*, *C albicans*, and *E coli*, all exhibiting multidrug resistance. During approximately 6 months of hospitalization, daily surgical consultations were conducted to enhance treatment with surgical debridement, dressing changes, and application of moist burn ointments. Despite these interventions, the pressure injury persisted without healing and progressively enlarged (as shown in Fig. 1A). Although chest CT scans and inflammatory markers indicated improvement in the pulmonary infection, the patient continued to experience recurrent, uncontrollable high fever. After half a year of complete standardized Western multimodal therapy, the patient still suffered from persistent unhealed wounds and intractable recurrent fever accompanied by obvious spleen-qi deficiency symptoms such as poor appetite, muscle atrophy, and sallow complexion. These refractory local and systemic manifestations served as the core indication for initiating systematic oral TCM intervention. On June 8, 2023, we initiated TCM treatment with the following prescription: Huangqi (Radix Astragali) 100 g, Renshen (Radix Ginseng) 30 g, Fuchao Baizhu (Rhizoma Atractylodis Macrocephalae) 30 g, Danggui (Radix Angelicae Sinensis) 20 g, Fuling (Poria) 30 g, Shengma (Rhizoma Cimicifugae) 15 g, Chaihu (Radix Bupleuri) 15 g, Chenpi (Pericarpium Citri Reticulatae) 15 g, Qinghao (Herba Artemisiae Annuae) 10 g, Chuanxiong (Rhizoma Chuanxiong) 15 g, Danshen (Radix Salviae Miltiorrhizae) 15 g, Longyanrou (Arillus Longan) 15 g, and Zhi Gancao (Radix Glycyrrhizae Preparata) 10 g. Decoct in water for nasogastric feeding, 1 dose daily, 150 mL ter in die (three times daily). After 4 days of treatment, the patient’s fever subsided and mental state improved. The original formula was modified by increasing Huangqi (Radix Astragali) to 150 g. Following 30 consecutive doses, the fever significantly diminished, the pressure injury gradually shrank, and the skin regained a rosy hue. However, phlegm production remained substantial. Therefore, the formula was modified by adding 20 g of Ziwan (Herba Asteris Tatarici) and 20 g of Baiqian (Rhizoma et Radix Cynanchi Stauntonii). After persisting with the herbal treatment for over 8 months, the patient’s pressure injury wound gradually developed a rosy hue, its depth decreased, the tongue coating became clean and dry, with no significant yellow purulent secretions adhering and no foul putrid odor detected (as shown in Fig. 1B). Although the patient still experienced intermittent fever during this period, it was mostly low-grade, and antibiotic use had been switched to intermittent administration. The pressure injury remained poorly healed for months. Although his fever had abated to some extent, intermittent febrile episodes persisted, requiring intermittent antibiotic therapy; the patient remained on sequential invasive mechanical ventilation without full weaning. To further explore TCM treatment, Professor Fang Bangjiang personally examined the patient at our hospital on February 22, 2024. At this time, the patient presented with the following symptoms: he presented with altered mental status and could not speak, yet he was able to slightly open his mouth and protrude his tongue on command. His complexion was sallow and wan, appetite poor, with copious white, viscous phlegm. He coughed weakly, experienced intermittent fever, and exhibited muscle atrophy and weakness in his limbs. The ulcerated area on his buttocks appeared pale and dry, with a moist tongue coating emitting a faint putrid odor. Urine was clear and copious, while stools were initially dry then loose. The tongue was swollen and pale, with a slightly thick white coating. The pulse was deep, fine, weak, and deficient (as shown in Fig. 2A). Specialist examination: blood pressure 114/70 mm Hg, heart rate 65 beats/min. The patient was alert but listless, with slightly delayed responses to commands. His complexion was sallow and icteric. His limb muscles were markedly wasted. He reported dyspnea upon minor exertion. Tracheostomy present; intermittent ventilator support used. Clear breath sounds bilaterally. Scattered rales audible in both lungs; no significant dry or wet rales heard. Regular heart rhythm. No pathological murmurs heard in cardiac auscultation areas. Abdomen soft with normal bowel sounds. No edema in lower extremities. Muscle strength approximately grade III in all limbs with normal muscle tone. Bilateral pathological signs negative. A stage IV pressure injury measuring approximately 8 cm × 10 cm was visible on the patient’s buttocks. The wound exhibited full-thickness tissue loss with exposure of deep structures including muscle and tendons. The wound bed contained pink granulation tissue, with areas of yellow necrotic tissue attached. Peripheral skin showed hyperpigmentation and maceration, without significant sinus tracts or subcutaneous fistulae. Supportive tests: Admission December 30, 2022: C-reactive protein, 114.5 mg/L; procalcitonin, 151.1 ng/mL; serum creatinine, 387 μmol/L; glycated hemoglobin, 9.7%; arterial blood gas: oxygenation index, 110 mm Hg; chest CT: bilateral pulmonary multiple infections. 2022 to 2023: Multiple bronchoalveolar lavage fluid cultures: *A baumannii* complex/*Acinetobacter hemolyticus*, *P aeruginosa*, *E coli*, *C albicans*, *Enterobacter cloacae*, *K pneumoniae*, all multidrug-resistant. Multiple sterile cultures from pressure injury exudate: *K pneumoniae*, *Staphylococcus pyogenes*, *C albicans*, *E coli*, all multidrug-resistant. Professor Fang’s Rounds (February 22, 2024) laboratory and imaging findings within 1 month: blood gas analysis: oxygenation index near 200 mm Hg; albumin: 30.50 g/L. Inflammatory markers, hepatic/renal function, cardiac enzymes, and heart failure markers trending toward normal. Chest CT revealed minimal pulmonary infection. No history of food or drug allergies.

**Figure 1.**
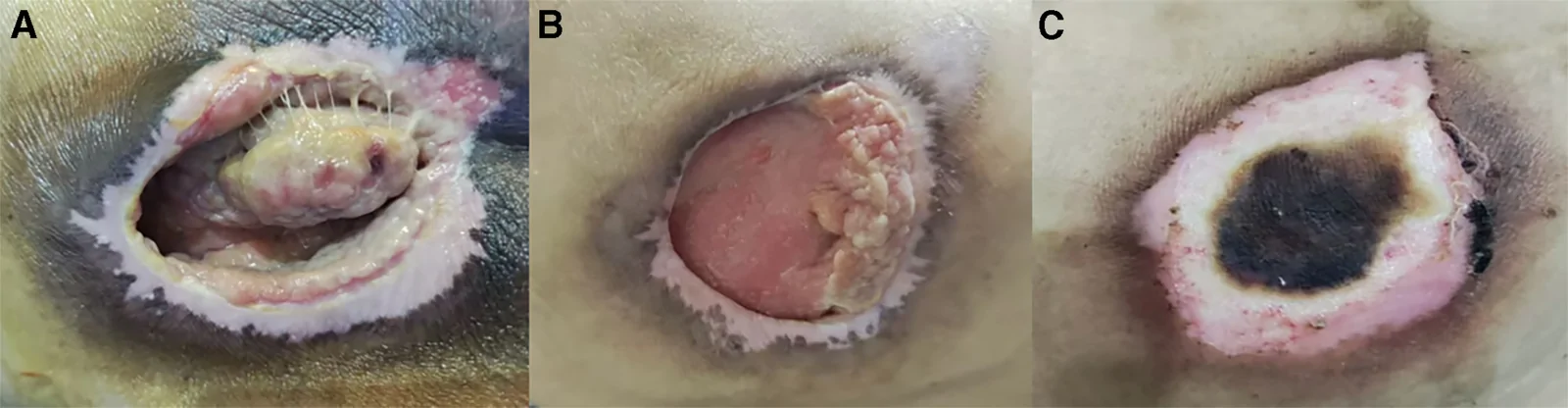
Healing process of stage IV pressure injury under integrated Chinese and Western medicine. (A) June 8, 2023: baseline wound after 6 months of Western treatment, 8 cm × 10 cm deep ulcer with exposed bone, heavy purulent discharge, and progressive deterioration before initial herbal treatment. (B) February 22, 2024: wound after 8 months of preliminary herbal decoction; exudate reduced but residual necrotic tissue remained, with persistent intermittent fever. (C) Mid-March 2024: complete wound healing after 20 days of Professor Fang’s optimized Modified Bu Zhong Yi Qi Tang.

**Figure 2.**
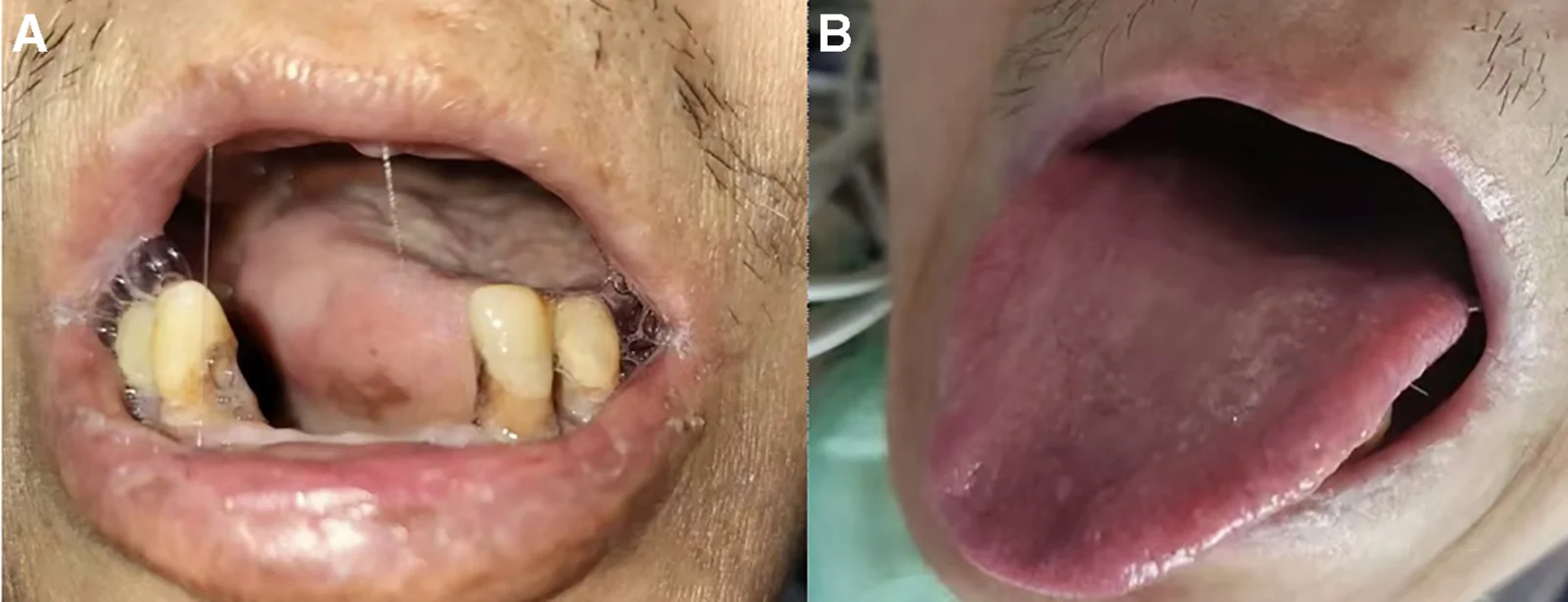
Tongue changes before and after optimized Modified Bu Zhong Yi Qi Tang. (A) February 22, 2024: swollen pale tongue with thick white greasy coating, indicating severe spleen–lung qi deficiency and phlegm-damp-toxin accumulation before optimized formula treatment. (B) Mid-March 2024: pale red tongue with thin moist coating, showing alleviated deficiency and damp stagnation after targeted herbal therapy.

### 2.2. TCM therapy

• TCM diagnosis: pressure injury; spleen deficiency with qi collapse, phlegm-stasis-damp-toxin obstruction syndrome.
• Treatment principle: Tonify the middle and boost qi, raise yang and lift collapse, support the interior and promote tissue regeneration.
• Chinese herbal formula: Modified Bu Zhong Yi Qi Tang. Specific ingredients: Huangqi (Radix Astragali) 150 g, Renshen (Radix Ginseng) 20 g, Baizhu (Rhizoma Atractylodis Macrocephalae) 30 g, Chaihu (Radix Bupleuri) 15 g, Shengma (Rhizoma Cimicifugae) 12 g, Zhi Baifuzi (Rhizoma Typhonii Preparatum) 24 g, Dan Nanxing (Arisaema Cum Bile) 30 g, Shu Dahuang (Radix et Rhizoma Rhei Preparatum) 9 g, Baijiangcao (Herba Patriniae) 10 g, Wugong (Scolopendra)2 g, Danggui (Radix Angelicae Sinensis) 15 g, Maidong (Radix Ophiopogonis) 30 g, Gan Shihu (Caulis Dendrobii) 30 g, Shu Dihuang (Radix Rehmanniae Preparata) 30 g, Jiegeng (Radix Platycodonis) 30 g, Lugen (Rhizoma Phragmitis) 30 g, Yiyiren (Semen Coicis)30 g, Qinghao (Herba Artemisiae Annuae) 30 g, and Sheng Gancao (Radix Glycyrrhizae) 18 g.

One dose daily, total 450 mL, taken in 3 divided doses half an hour after breakfast, lunch, and dinner. Continue for over 20 days.

The core pathogenesis of this patient was severe spleen deficiency with sunken qi, accompanied by accumulated phlegm, blood stasis, and damp-toxin. Single external wound-healing formulas or heat-clearing detoxifying prescriptions only relieve superficial symptoms and cannot improve the patient’s persistent visceral deficiency, which is the root cause of unhealed ulcers. Modified Bu Zhong Yi Qi Tang was selected as the core regimen because it targets tonifying middle-jiao qi and lifting sunken yang to treat the fundamental deficiency; compatible supplementary herbs were added to resolve phlegm, remove stasis, and clear damp-toxin for symptomatic management, realizing simultaneous treatment of root and branch lesions.

### 2.3. Treatment outcomes

After receiving the modified herbal formula for over 20 consecutive days, the patient’s mental state improved markedly; he could follow simple motor commands, with better coordination for mouth opening and tongue protrusion. His complexion turned pale yellow with a lustrous appearance. Sputum production decreased significantly, appearing white and thin in consistency, and the patient could autonomously cough up small amounts of sputum. Muscle weakness in the limbs lessened, allowing for slight limb movement with muscle strength at grade IV. Appetite increased, with nasogastric feeding reaching 200 mL per session. Bowel movements and urination were normal. The tongue appeared pale red with a thin white coating, moderately moist. The pulse was deep, fine, and forceful (as shown in Fig. 2B). The pressure injury showed a pale red color, with surrounding redness and swelling subsiding. No exudate was present on the ulcer surface, and no foul odor was detected. The ulcer had essentially healed completely (as shown in Fig. 1C). The patient remained fever-free, successfully discontinued mechanical ventilation, and ceased antibiotic therapy. Subsequently, the patient continued rehabilitation treatment at our hospital and was discharged after prolonged inpatient care with improved condition. We regularly monitored liver and renal function, routine blood tests, and vital signs throughout herbal therapy to screen adverse reactions and herb–drug interactions. No adverse events or obvious drug–herb interactions occurred.

To clarify the chronological sequence of multiple overlapping interventions throughout hospitalization, we summarize all key clinical events in the timeline below (Table 1).

**Table 1:** Timeline of key clinical and therapeutic events.

Time point	Detailed clinical and therapeutic interventions
December 30, 2022	Admitted with severe pneumonia and transferred to ICU; ventilation, long-term antibiotics, debridement, and enteral nutrition started
June 8, 2023	TCM decoction initiated after 6 months of ineffective Western treatment. Fever and mental status improved on day 4; Astragali Radix was increased. After 8 months of herbal adjustment, the wound got cleaner and shallower, but intermittent low fever and ventilation dependence remained
February 22, 2024	Professor Fang’s ward rounds; formal Modified Bu Zhong Yi Qi Tang treatment began
Mid-March 2024	The ulcer nearly healed after 20 days of adjusted decoction; ventilation and antibiotics were stopped, followed by pulmonary rehabilitation
March 2024–discharge	Combined oral TCM decoction and pulmonary rehabilitation until discharge

ICU = intensive care unit, TCM = traditional Chinese medicine.

## 3. Discussion

This case reports on a long-stay critically ill ICU patient with refractory stage IV pressure injury, recurrent multidrug-resistant bacterial pneumonia, and persistent ventilator dependence, which represents a common and challenging clinical scenario in critical care practice. After 6 months of standardized Western treatment, including surgical debridement, long-term broad-spectrum antibiotics, routine pressure-relief care, and enteral nutrition, the patient’s wound continued to deteriorate with persistent recurrent fever. This observational case provides real-world evidence of integrated Chinese and Western therapy for critical illness-related refractory pressure injury, offering practical reference for the clinical management of similar complicated intractable wounds.

In TCM theory, prolonged local compression causes qi stagnation and blood stasis, blocks meridian circulation, and reduces qi-blood perfusion to local tissues, ultimately leading to tissue necrosis and pressure injury formation.^[7]^ Ancient TCM literature documented that chronically debilitated patients are prone to pressure injuries due to persistent qi-blood depletion, damp-heat invasion, and toxin accumulation.^[8]^ TCM management of pressure injuries includes internal and external interventions: external therapies prioritize heat-clearing, detoxification, and granulation promotion, while internal treatments focus on qi tonification, blood activation, and debridement.^[9]^ Based on years of critical illness experience, Professor Fang Bangjiang proposed the “acute deficiency syndrome” theory, which well explains the present patient’s disease progression characterized by acute healthy qi exhaustion, chronic visceral impairment, and simultaneous root deficiency and superficial excess. Severe exogenous heat pathogen rapidly consumed pulmonary qi and yin, progressing to type I respiratory failure requiring invasive ventilation. Sustained multidrug-resistant infection, recurrent high fever, tracheotomy, and long-term immobilization induced high catabolism and low bodily reserve, forming the fundamental deficient basis for pressure injury development. Long-term mechanical ventilation and impaired oral intake further damaged spleen–stomach transportation, resulting in typical spleen deficiency with sunken qi. Deficient spleen qi failed to nourish skin and muscles, while impaired lung qi weakened systemic qi-blood circulation. Persistent buttock compression aggravated local meridian stasis, and the interplay of deficiency and stasis finally caused deep stage IV bone-exposed ulcers. Wound purulent discharge, multidrug-resistant pathogens, and copious sticky sputum indicated superficial phlegm-stasis-damp-toxin accumulation, whereby deficient healthy qi failed to eliminate pathogenic toxins and led to unhealed and progressive ulcers. Even after partial control of pulmonary infection via Western therapy, intermittent fever persisted, which was differentiated as deficiency-induced internal heat derived from lung-spleen qi deficiency and floating deficient yang, rather than excess heat.^[10]^ Long-term spleen–lung weakness impaired qi-blood generation, resulting in limb atrophy, poor sputum expectoration, and recurrent deficiency-type fever.

Conventional Western interventions contributed synergistically to the patient’s overall improvement during TCM treatment. Regular wound debridement and daily dressing changes reduced necrotic tissue and local bacterial load, and targeted intermittent antibiotics controlled multidrug-resistant infection. Optimized high-protein enteral nutrition corrected hypoalbuminemia and provided substrates for tissue repair, while standardized pressure-relief nursing continuously reduced mechanical compression on the wound. These supportive Western measures created favorable conditions for gradual wound recovery during long-term integrated therapy. Targeting the patient’s core pathogenesis of spleen–lung qi deficiency combined with phlegm-stasis-damp-toxin obstruction, Modified Bu Zhong Yi Qi Tang was adopted as the core regimen. Different from simple external ointments or single heat-clearing formulas that only relieve superficial symptoms, this formula tonifies middle-jiao qi and lifts sunken yang to treat the fundamental deficiency, while resolving phlegm, unblocking stasis, and clearing damp-toxin to improve local lesions, achieving root and symptom dual regulation. In this formula, Huangqi (Radix Astragali) was used as the monarch herb to tonify primordial qi, support toxin excretion, and promote tissue regeneration.^[11]^ Renshen (Radix Ginseng) and Baizhu (Rhizoma Atractylodis Macrocephalae) served as minister herbs to strengthen spleen transportation, improve appetite and defecation, and replenish the source of qi-blood generation. Chaihu (Radix Bupleuri) and Shengma (Rhizoma Cimicifugae) lifted sunken yang and guided tonifying herbs to promote qi-blood perfusion to wound tissues. Dan Nanxing (Arisaema Cum Bile) and Zhi Baifuzi (Rhizoma Typhonii Preparatum) eliminated obstructive phlegm turbidity; Baijiangcao (Herba Patriniae) and Shu Dahuang (Radix et Rhizoma Rhei Preparatum) cleared abscess toxin and drained internal heat. Wugong (Scolopendra) unblocked stagnant meridians to improve local wound microcirculation, while Danggui (Radix Angelicae Sinensis) nourished and activated blood. Maidong (Radix Ophiopogonis), Gan Shihu (Caulis Dendrobii), and Shu Dihuang (Radix Rehmanniae Preparata) supplemented consumed yin fluid and balanced the warm-dry property of qi-tonifying herbs. Jiegeng (Radix Platycodonis), Lugen (Rhizoma Phragmitis), and Yiyiren (Semen Coicis) resolved phlegm, drained damp pus, and relieved deficiency heat, while Qinghao (Herba Artemisiae Annuae) further alleviated lingering tidal fever. Sheng Gancao (Radix Glycyrrhizae) harmonized all herbal ingredients and protected the spleen–stomach foundation. During combined multimodal treatment, changes potentially related to herbal intervention were observed, including improved visceral function, ameliorated systemic deficiency, and reduced phlegm-toxin stasis, accompanied by gradual wound granulation, decreased purulent secretions, and resolution of fever. Given the single observational case design without a control group, no exclusive causal relationship between TCM medication and wound healing can be concluded; clinical improvement was achieved via comprehensive multimodal integrated Chinese and Western care.

Consistent with existing clinical studies and case series, existing literature suggests potential clinical benefit from integrated Chinese-Western approaches for critical illness-complicated refractory pressure injuries compared with Western monotherapy.^[6]^ Most published studies focus on external TCM ointments for common bedridden patients, while few reports address oral tonifying formulas for severe stage IV pressure injuries combined with multidrug-resistant infection and long-term ventilator dependence in ICU patients.^[12,13]^ This case complements current literature by describing a favorable clinical outcome following the addition of internal TCM tonifying intervention to multimodal care for extremely complicated critical pressure injuries, further providing observational clinical insight into qi-tonifying and spleen-strengthening TCM strategies for consumptive critical illness.

Several limitations of this single-case report should be acknowledged. First, this is a single observational study without a control group, precluding quantitative efficacy comparison. Second, multiple concurrent interventions including wound care, antibiotics, nutritional support, standardized nursing management, and TCM herbal treatment were applied simultaneously, making it impossible to independently distinguish and quantify the unique contribution of TCM therapy. Third, the patient’s severe critical illness status, individualized pathogen spectrum, and long-term ventilator dependence limit the generalizability of these findings. Fourth, no long-term follow-up data were collected, so sustained therapeutic efficacy and wound recurrence risk cannot be evaluated.

## 4. Conclusion

Patients in the ICU usually suffer from severe primary diseases. Pressure injuries developing in patients with deficient vital qi are often protracted and hard to heal. Based on TCM holistic thinking and syndrome differentiation, this paper reports a clinical observation of Modified Bu Zhong Yi Qi Tang guided by the principles of tonifying the middle-jiao, lifting sunken yang, and consolidating root to promote granulation. This single favorable case cannot serve as definitive evidence of general therapeutic efficacy. The patient’s deep refractory pressure injury failed to improve under routine Western wound care. After 20 days of combined Modified Bu Zhong Yi Qi Tang and standardized Western interventions including debridement, antibiotics, and wound nursing, obvious granulation growth and accelerated necrotic tissue shedding were observed, which promoted wound repair. The positive recovery arose from integrated Chinese-Western management, so this isolated case cannot confirm the independent efficacy of the herbal formula. This report is limited to a single patient, and the encouraging outcome cannot be generalized to all ICU patients with severe pressure injuries. Further large-sample prospective studies and controlled clinical trials are required to verify the clinical value of TCM herbal therapy for pressure injuries.

## Acknowledgments

All authors would like to express their gratitude to the patient mentioned in this article for allowing us to report this case.

## Author contributions

**Writing – original draft:** Dan Liao, Yan Zhang.

**Writing – review & editing:** Dan Liao, Yan Zhang.

**Methodology:** Bangjiang Fang, Yan Zhang.

**Project administration:** Bangjiang Fang, Yan Zhang.

**Conceptualization:** Jianjian Li, Jing Wang.

**Funding acquisition:** Jianjian Li, Yan Zhang.

**Investigation:** Jianjian Li.

**Data curation:** Jing Wang, Wei He, Xiaosong Lyu.

**Formal analysis:** Jing Wang, Wei He.

**Resources:** Yan Zhang.
